# Content-based histopathology image retrieval using CometCloud

**DOI:** 10.1186/1471-2105-15-287

**Published:** 2014-08-26

**Authors:** Xin Qi, Daihou Wang, Ivan Rodero, Javier Diaz-Montes, Rebekah H Gensure, Fuyong Xing, Hua Zhong, Lauri Goodell, Manish Parashar, David J Foran, Lin Yang

**Affiliations:** Department of Pathology and Laboratory Medicine, Rutger Robert Wood Johnson Medical School, 675 Hoes Lane, Piscataway, NJ USA; Center for Biomedical Imaging and Informatics, Rutgers Cancer Institute of New Jersey, New Brunswick, NJ USA; Rutgers Discovery Informatics Institute and NSF Cloud and Autonomic Computing Center, Department of Electrical and Computer Engineering, Rutgers University, Piscataway, NJ USA; Department of Radiology, Rutgers - Robert Wood Johnson Medical School, Piscataway, NJ USA; Division of Biomedical Informatics, Department of Biostatistics, Department of Computer Science, University of Kentucky, Lexington, KY USA

**Keywords:** Histopathology, Digital pathology, Content-based image retrieval, High performance computing

## Abstract

**Background:**

The development of digital imaging technology is creating extraordinary levels of accuracy that provide support for improved reliability in different aspects of the image analysis, such as content-based image retrieval, image segmentation, and classification. This has dramatically increased the volume and rate at which data are generated. Together these facts make querying and sharing non-trivial and render centralized solutions unfeasible. Moreover, in many cases this data is often distributed and must be shared across multiple institutions requiring decentralized solutions. In this context, a new generation of data/information driven applications must be developed to take advantage of the national advanced cyber-infrastructure (ACI) which enable investigators to seamlessly and securely interact with information/data which is distributed across geographically disparate resources. This paper presents the development and evaluation of a novel content-based image retrieval (CBIR) framework. The methods were tested extensively using both peripheral blood smears and renal glomeruli specimens. The datasets and performance were evaluated by two pathologists to determine the concordance.

**Results:**

The CBIR algorithms that were developed can reliably retrieve the candidate image patches exhibiting intensity and morphological characteristics that are most similar to a given query image. The methods described in this paper are able to reliably discriminate among subtle staining differences and spatial pattern distributions. By integrating a newly developed dual-similarity relevance feedback module into the CBIR framework, the CBIR results were improved substantially. By aggregating the computational power of high performance computing (HPC) and cloud resources, we demonstrated that the method can be successfully executed in minutes on the Cloud compared to weeks using standard computers.

**Conclusions:**

In this paper, we present a set of newly developed CBIR algorithms and validate them using two different pathology applications, which are regularly evaluated in the practice of pathology. Comparative experimental results demonstrate excellent performance throughout the course of a set of systematic studies. Additionally, we present and evaluate a framework to enable the execution of these algorithms across distributed resources. We show how parallel searching of content-wise similar images in the dataset significantly reduces the overall computational time to ensure the practical utility of the proposed CBIR algorithms.

## Background

A growing number of leading institutions now routinely utilize digital imaging technologies to support investigative research and routine diagnostic procedures. The exponential rate at which images and videos are being generated has resulted in a significant need for efficient content-based image retrieval (CBIR) methods, which allow one to quickly characterize and locate images in large collections based upon the features of a given query image. CBIR has been one of the most active research areas in a wide spectrum of imaging informatics fields over the past few decades [1–13]. Several domains stand to benefit from the use of CBIR including cinematography, education, investigative basic and clinical research, and the practice of medicine. CBIR has been successfully utilized in applications spanning radiology [4, 11, 14, 15], pathology [9, 16–18], dermatology [19, 20], and cytology [21–23].

There have been several successful CBIR systems that have been developed for medical applications since the 1980’s. Several approaches utilize simple features such as color histograms [24], shape [4, 22], texture [6, 25], or fuzzy features [7] to characterize the content of images while allowing higher level diagnostic abstractions based on systematic queries [4, 25–27]. The recent adoption and popularity of case-based reasoning [28] and evidence-based medicine [29] has created a compelling need for more reliable image retrieval strategies to support diagnostic decisions. In fact, a number of state-of-the-art CBIR systems [4, 9, 11–13, 15, 16, 25, 30–32] have been designed to support the processing of queries across imaging modalities.

With the advent of whole-slide imaging technology, the size and scale of image-based data has grown tremendously, making it impractical to perform matching operations across an entire image dataset using traditional methods. To meet this challenge, a new family of strategies are being developed, which enable investigators to perform sub-region searching to automatically identify image patches that exhibit patterns that are consistent with a given query patch. In practice, this approach makes it possible to select a region or object of interest within a digitized specimen as a query while the algorithm systematically identifies regions exhibiting similar characteristics in either the same specimen or across disparate specimens. The results can then be used to draw comparisons among patient samples in order to make informed decisions regarding likely prognoses and most appropriate treatment regimens.

To perform a region-of-interest (ROI) query, Vu et al. [33] presented a Sam Match framework-based similarity model. The use of a part-based approach was later reported in [34] to solve the CBIR problem by synthesizing a DoG detector, and a local hashing table search algorithm. The primary limitation of this approach, however, was the time cost of the large number of features that need to be computed. Intra-expansion and inter-expansion strategies were later developed to boost the hash-based search quality based on a bag-of-features model which could more accurately represent the images. Recently, a structured visual search method was developed to perform CBIR in medical image datasets [35]. The primary advantage of this framework is that it is flexible and can be quickly extended to other modalities.

Most CBIR algorithms rely on content localization, feature extraction, and user feedback steps [5–7, 25, 27, 36–40]. The retrieved results are then ranked by some criteria, such as appearance similarity or diagnostic relevance, which can also serve as a measure of the practical usability of the algorithm. Typically the retrieved images only include those cases with the most similar appearance to a given query image whereas introducing relevance feedback [41–47] to CBIR provides a practical means for addressing the semantic gap between visual and semantic similarity.

Large-scale image retrieval applications are generally computationally expensive. In this paper, we present the use of the CometCloud [48, 49] to execute CBIR in a parallel fashion on multiple high performance computing (HPC) and cloud resources as a means for reducing computational time significantly. CometCloud is an autonomic cloud framework that allows dynamic, on-demand federation of distributed infrastructures. It also provides an effective programming platform that supports MapReduce, Workflow, and Master-Worker/BOT models making it possible for investigators to quickly develop applications that can run across the federated resources [49–53]. The algorithm that our team developed exploits the parallelism of CBIR by combining the HPC assets at Rutgers University with external cloud resources. Moreover, our solution uses cloud abstractions to federate resources elastically to achieve acceleration, while hiding infrastructure and deployment details. In this way, the CBIR algorithm can be made available as accessible services to end users.

The contributions of this paper are: 1) a novel CBIR algorithm based on a newly developed coarse-to-fine searching criteria which is coupled with a novel feature called hierarchical annular histogram (HAH); 2) a CBIR refinement schema based on dual-similarity relevance feedback; and 3) a reliable parallel implementation of the CBIR algorithm based on Cloud computing.

## Methods

### Research design

After discussing the needs and requirements of pathologists from their perspective, the CBIR study is designed to quickly and accurately find images exhibiting similar morphologic and staining characteristics throughout a single or collection of imaged specimens. Our team specifically choose to use Giemsa stained peripheral blood smear and hematoxylin and eosin (H&E) stained renal glomeruli datasets to systematically test the algorithms since these are two routine use case scenarios that our clinical colleagues indicated might benefit from the proposed technology. Leukocytes are often differentiated based on traditional morphological characteristics, however the subtle visible differences exhibited by some lymphomas and leukemias result in a significant number of false negative during routine screenings. In many cases, the diagnosis is only rendered after conducting immunophenotyping and a range of other molecular or cytogenetic studies. The additional studies are expensive, time consuming, and usually require fresh tissues that may not be readily available [54]. Pre-transplantation biopsies of kidney grafts have become a routine means for selecting organs which are suitable for transplantation from marginal donors. The main histopathology characteristics that are routinely evaluated by pathologists are percentage of glomerulosclerosis, interstitial fibrosis, and degree of vascular pathology [55]. The central incentive for developing the CBIR algorithms is to determine a reliable means for assisting pathologists when they are called upon to render diagnostic decisions based on whole-slide scanned specimens.

In this paper, we present a novel content-based image retrieval (CBIR) algorithm that is systematically tested on both imaged Giemsa stained peripheral blood smears and digitized H&E stained renal glomeruli specimens. Because of the intense computational requirements of the algorithms, our team systematically investigate the use of high performance computing solutions based on CometCloud to distribute the tasks of performing CBIR to significantly reduce the overall running time. The details of datasets, the relevant CBIR algorithms, and the CometCloud implementation of the methods are explained in detail in the following sections.

In the case of Giemsa stained peripheral blood smear datasets, the algorithms operate on a given query patch to quickly and reliably detect other leukocytes of the same class throughout the imaged specimen in support of diagnostic decisions. These hematopathology datasets were acquired using a 20× objective to provide a gross overview of the specimen while also supplying sufficient resolution to distinguish among different classes of leukocytes. The dataset consisted of 925 imaged blood smears (1000×1000 pixels). In the case of the H&E stained renal glomeruli datasets, the algorithms are used to process any given query patch to discriminate necrotic glomeruli and normal glomeruli throughout imaged kidney tissue specimens. In these experiments, our team cropped 32 images (5024×3504 pixels) from within eight whole-slide renal specimens using a 20× objective.

Quality control of all datasets was conducted by an experienced pathologist (Dr. Zhong) whereas query image patches and ground-truth classification were determined by two pathologists (Dr. Zhong and Dr. Goodell). The retrieved results were evaluated by both pathologists through a completely independent and blinded process. During the peripheral blood smear experiments, pathologists were asked to assign each leukocyte retrieved using the CBIR algorithm to either the relevant or non-relevant class as a means for judging the appropriateness of each returned patch. In all, there were five different classes of leukocytes used in the studies. During the renal glomeruli studies, either a relevant or non-relevant assignment was made to judge the performance of the algorithms in distinguishing between necrotic glomeruli and normal glomeruli.

**Figure 1:**
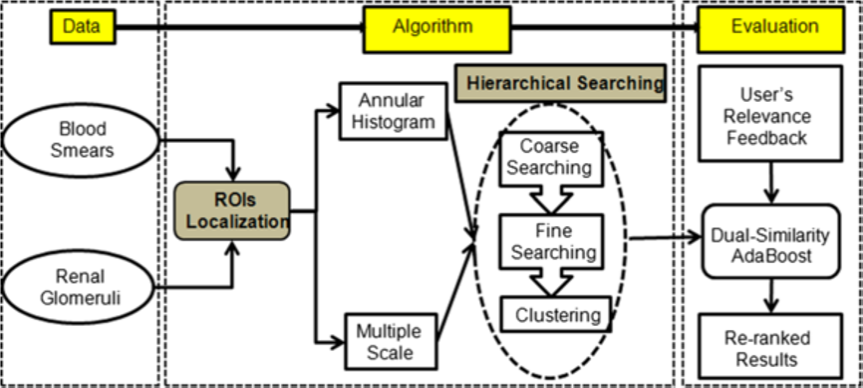
**Workflow of the proposed CBIR algorithm.**

The CBIR algorithms consist of four major steps: 1) regions of interest (ROIs) localization, 2) hierarchical three-stage searching, 3) retrieval refinement based on dual-similarity relevance feedback, and 4) high performance computing using CometCloud [48]. Figure 1 illustrates the actual workflow of the process.

### Step 1: regions of interest localization

The first step is to locate the regions of interest (ROIs) throughout the imaged specimens by excluding the background regions from the candidate objects. Using color-decomposition and morphology [56] based preprocessing, the algorithm identifies application-specific ROIs. These regions serve as candidate searching regions in the subsequent stages of hierarchical searching. Candidate image patches are generated using a sliding window approach with an overlapping ratio within the range of [ 50*%*,90*%*].

### Step 2: hierarchical three-stage searching

The hierarchical three-stage searching method includes: coarse searching, fine searching, and mean-shift clustering.

#### Coarse searching

**Figure 2:**
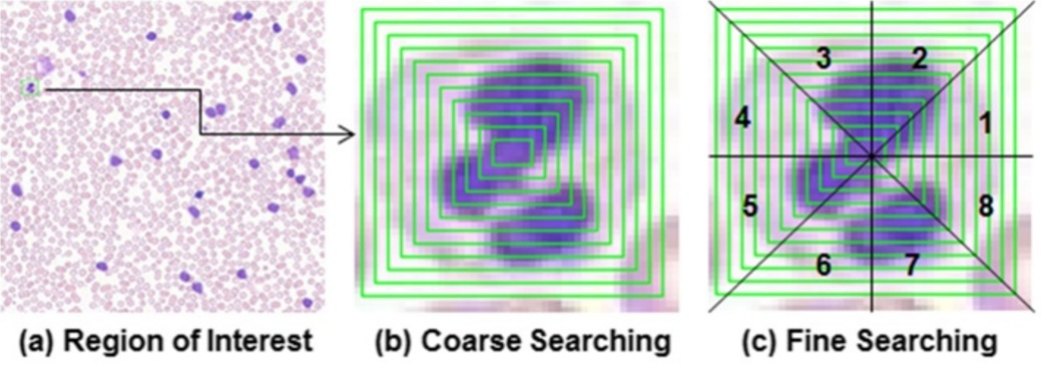
**An illustration of the hierarchical searching framework: (a) region of interest, (b) coarse searching step, and (c) fine searching step.**

Let *Q* represents a query image patch and *P* serves the candidate image patches. Each patch is divided into consecutive concentric rectangular bin regions (termed as rings) as shown in Figure 2(a-b). As the number of rings, *r*, increases, more detailed image characteristics are captured and while the computational time increases accordingly. *r* is determined based on cross-validation. Figure 2(b) illustrates the process of coarse searching. Given a query image patch, the algorithm computes local features from the innermost ring. Based on a similarity measure between candidate image patches, *P*, and the query image, *Q*, retrieved image patches, *P*, are ranked from high to low, and only the top 50*%* ranked candidates are reserved at each step. This procedure continues until the outermost ring is reached. This cascade structure significantly reduces the computational time, as 50% of the image patches are eliminated in the very first stage of processing by simply evaluating features in the innermost ring.

#### Fine searching

After the coarse searching stage has been completed, each rectangular annular ring from both the query and candidate patches are equally subdivided into eight segments, and local features are calculated in each segment. The final candidates are chosen based on a similarity measure of a concatenated feature vector corresponding to the eight segments. Figure 2(c) illustrates the process of the fine searching. This stage is designed to capture the spatial configuration of the local features. Due to the limited number of candidates passing through the coarse searching stage, the computational time for completing this stage is dramatically reduced.

#### Mean-shift clustering

In order to assemble the final retrieval results, mean-shift (MS) clustering [57] is applied to the top ranked candidate patches, which have survived both the coarse and fine searching stages. The bandwidth *b* for the mean-shift clustering is calculated as , where *w* is the width of the query image and *h* is the height of the query image. In this way, the final CBIR results are obtained.

### HAH Feature and feature comparison

#### HAH feature

To implement the hierarchical searching framework, we develop a hierarchical annular histogram (HAH). The intensity color histograms of consecutive concentric rectangular rings are calculated and concatenated together to form a coarse searching feature vector, *H*^*c*^=(*h*_1_,*h*_2_,…,*h*_*r*_), where *h*_*i*_ is the intensity color histogram of the *i*th ring, *i*∈ [ 1,*r*] and *r* is the number of rings selected for the HAH feature. For fine searching, each rectangular annular ring is equally divided into eight segments, and the color histogram is calculated from each segment sequentially and then concatenated together to form the fine searching feature vector, *H*^*f*^=(*h*_1,1_,…,*h*_1,8_,*h*_2,1_,…,*h*_2,8_,…,*h*_*r*,1_,…,*h*_*r*,8_), where *h*_*i*,*j*_ is the intensity color histogram of the *i*th ring within the *j*th segment, *j*∈ [ 1,8]. Here superscript *c* represents coarse searching and *f* represents fine searching. Throughout the CBIR study, we use Euclidean distance as the similarity measure. The distance *D*_*i*_, between the *i*th candidate patch *v*_*i*_ and the query patch *q* in coarse searching and fine searching are defined as  and , respectively:


where . Here *H*^*c*^(*q*^*c*^),*H*^*f*^(*q*^*f*^) are the feature vector of query image during coarse searching and fine searching stages, respectively, and  are the feature vector of the *i*th candidate patch in the coarse searching and fine searching stages, respectively.

**Figure 3:**
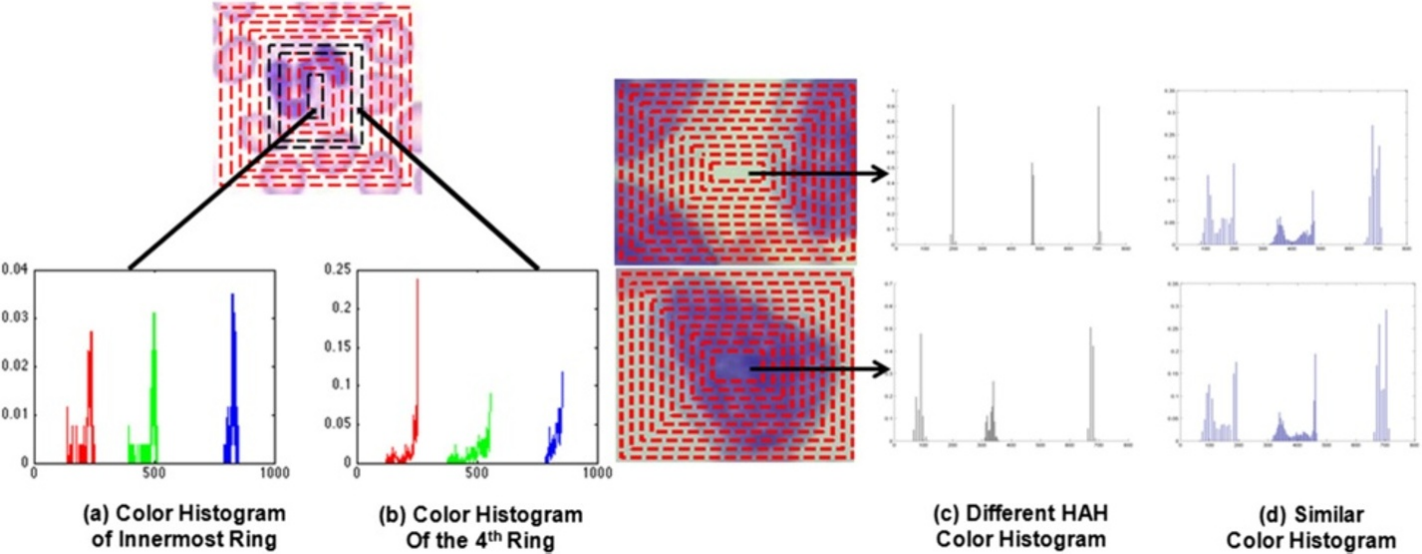
**An illustration of HAH calculation. (a)** Color histogram of the central ring. **(b)** Color histogram of the fourth ring from the center. An example of two patches with **(c)** different HAH, but **(d)** similar color histogram of the entire image.

Figure 3(a) and (b) illustrate the calculation of the HAH from the innermost rectangle and the fourth ring from the center. Figure 3(c) and (d) show an example of two image patches with similar traditional color histogram (d), but completely different HAH (c). This demonstrates the capacity of the HAH to differentiate among image patches exhibiting similar total color distributions, but different spatial configurations.

In order to compare the performance of the HAH feature in CBIR, the Gabor wavelet feature [58] and co-occurrence texture feature [59, 60] were compared with the HAH feature with respect to both speed and accuracy using both imaged peripheral blood smear and renal glomeruli datasets. For the purpose of the studies, precision and recall were used to measure the performance of the CBIR algorithm. Precision is defined as the ratio between the number of retrieved relevant images and the total number of retrieved images. Recall is defined as the ratio between the number of retrieved relevant images and the total number of relevant images in the datasets.

#### The Gabor wavelet feature

The Gabor wavelet feature is used to describe the image patterns at a range of different directions and scales. Throughout the experiments, we utilize a Gabor filter with 8 directions and 5 scales, (*M*=5,*N*=8), and the mean value and standard deviation of each filtered image are concatenated to form a feature vector: *f*=(*μ*_1,1_,*σ*_1,1_,*μ*_1,2_,*σ*_1,2_,…,*μ*_5,8_,*σ*_5,8_), in which *μ*_*m*,*n*_ and *σ*_*m*,*n*_ represent the mean value and standard deviation of the filtered image using Gabor filter at the *m*th scale and *n*th direction, *m*∈ [ 1,*M*],*n*∈ [ 1,*N*]. The distance *D*_*i*_ between the *i*th candidate patch *v*_*i*_, and the query patch *q*, is defined as


where .

#### COOC texture feature

Co-occurrence (COOC) matrices, also called spatial gray-level dependence matrices, were first proposed by Haralick et al. [59, 60]. COOC matrices are calculated from an estimation of the second-order joint conditional probability of the image intensity with various distances and four specific orientations (0^0^, 45^0^, 90^0^, 135^0^). COOC texture feature using the COOC matrices quantifies the distribution of gray-level values within an image. For the feature comparison experiment, COOC texture feature including contrast, correlation, energy, and homogeneity [60], is calculated from the COOC matrices within the candidate ROIs and the query image. The distance, *D*_*i*_, between the *i*th candidate patch *v*_*i*_, and the query patch *q*, is defined as


where , and *F* = {contrast, correlation, energy, homogeneity}.

### Stage 3: CBIR retrieval refinement using a dual-similarity relevance feedback

Relevance feedback is an interactive procedure which is used to refine the initial retrieval results. Upon completion of the initial retrieval, top ranked retrieval images were reviewed by two pathologists with consensus to label them as relevant or non-relevant as previously described. These responses are used as users’ feedback to re-rank the retrieval results accordingly.

Two types of similarities are used in the above retrieval and feedback procedure: similarity in visual appearance as measured by image feature distance and similarity in semantic category as measured as relevant or non-relevant. Current relevance feedback algorithms typically only consider the second similarity. In our algorithm, we develop a dual-similarity schema that combines both types of similarity measures. This is achieved by rebuilding the initial distributions of training samples in an on-line manner.

For each top ranked retrieved image, a 256×3×*r* dimension feature vector is constructed, where *r* is the number of rings defined in the hierarchical searching process. Dimension reduction using principal component analysis (PCA) is applied to the original HAH feature space, and the top principal components accounting for 90% of the total variance are used as inputs for the following relevance feedback procedure.

Adaboost [61] is utilized to train an ensemble classifier composed of a set of weak learners. Given a training dataset, a strong classifier is built as a weighted sum of weak learners by minimizing the misclassification errors. Define weight *W*_*i*_, to be measured by a normalized Euclidean distance *D*_*i*_, representing the image appearance similarity between a pair of retrieved image and the original query. The initial distribution of the training samples is recalculated to update the classifier to place more weights on the visually similar cases following the relevance feedback step. The algorithm is summarized as follows.


### Step 4: accelerating CBIR using CometCloud

Due to the data-independence property of the CBIR algorithm, we can formulate our problem as a set of heterogeneous and independent or loosely couple tasks. In this way, we can parallelize and solve our problem using the aggregated computational power of distributed resources. Our team has designed and developed a framework that enables the execution of CBIR across distributed, federated resources. Our framework uses cloud abstractions to present the underlying infrastructure as a single elastic pool of resources regardless of their physical location or specific particularities. In this way, computational resources are dynamically provisioned on-demand to meet the application’s requirements. These resources can be high performance computing grids, clouds, or supercomputers. In the current application, the framework is built on top of CometCloud [48]. CometCloud is purposely chosen for this application since it enables dynamic and on-demand federation of advanced cyber-infrastructures (ACIs). It also provides a flexible application programming interface (API), for developing applications that can take advantage of federated ACIs. Furthermore, it provides fault-tolerance in the resulting infrastructure.

The framework used to run the CBIR algorithm across federated resources is implemented using the master/worker paradigm. In this scenario, the CBIR software serves as a computational engine, while CometCloud orchestrates the entire execution. The master/worker model is suitable for problems with a large pool of independent tasks, where both the tasks and the resources are heterogeneous. Using this approach, the master component generates tasks, collects results, and verifies that tasks are properly executed. Each task contains the description of the images to be processed. All tasks are automatically placed in the CometCloud-managed distributed task space for execution. Workers are dedicated to carry out tasks pulled from the CometCloud task space and send results back to the master.

The implementation that we have presented has several important and highly desirable properties. From the user’s perspective, the framework creates a cloud abstraction on top of the resources that hides infrastructure details and offers the CBIR software as a readily accessible service. In this way, one can query the database using different algorithms via a simple interface without consideration of how and where queries are executed. On the other hand, from the developer’s perspective, the integration of the existing CBIR software with the CometCloud framework does not require any adjustments on the application side. Additionally, the resulting framework completely operates within the limits of the end-user space. This means that it is possible to aggregate computational resources without special privileges, which is very important when using external resources.

## Results and discussion

### CBIR results and feature comparison

**Figure 4:**
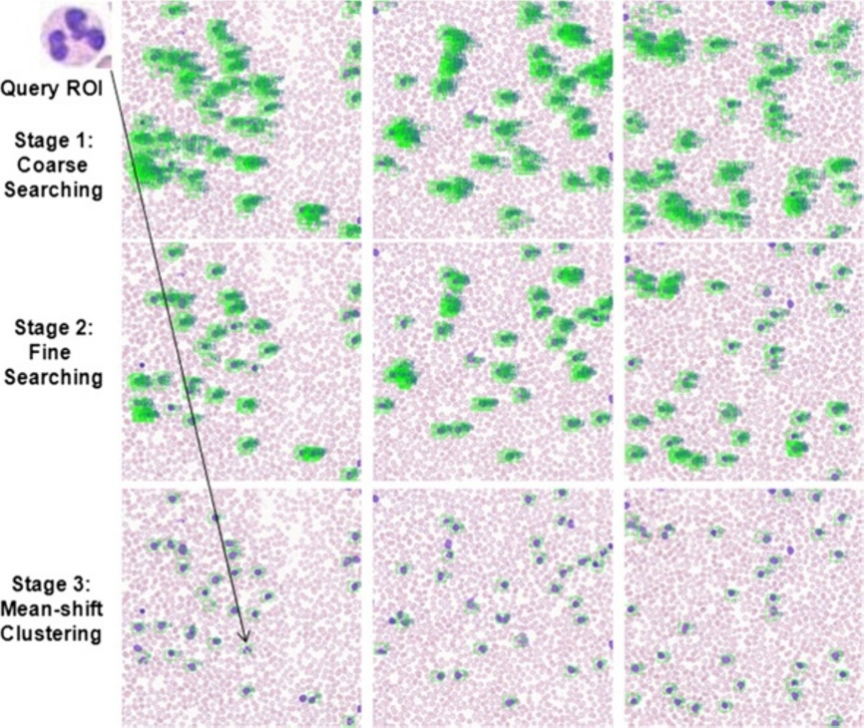
**An illustration of results of the three-stage CBIR searching using one neutrophil as a query image from peripheral blood smears acquired at 20× objective, in which green box labeled regions represent the candidate patches.**

**Figure 5:**
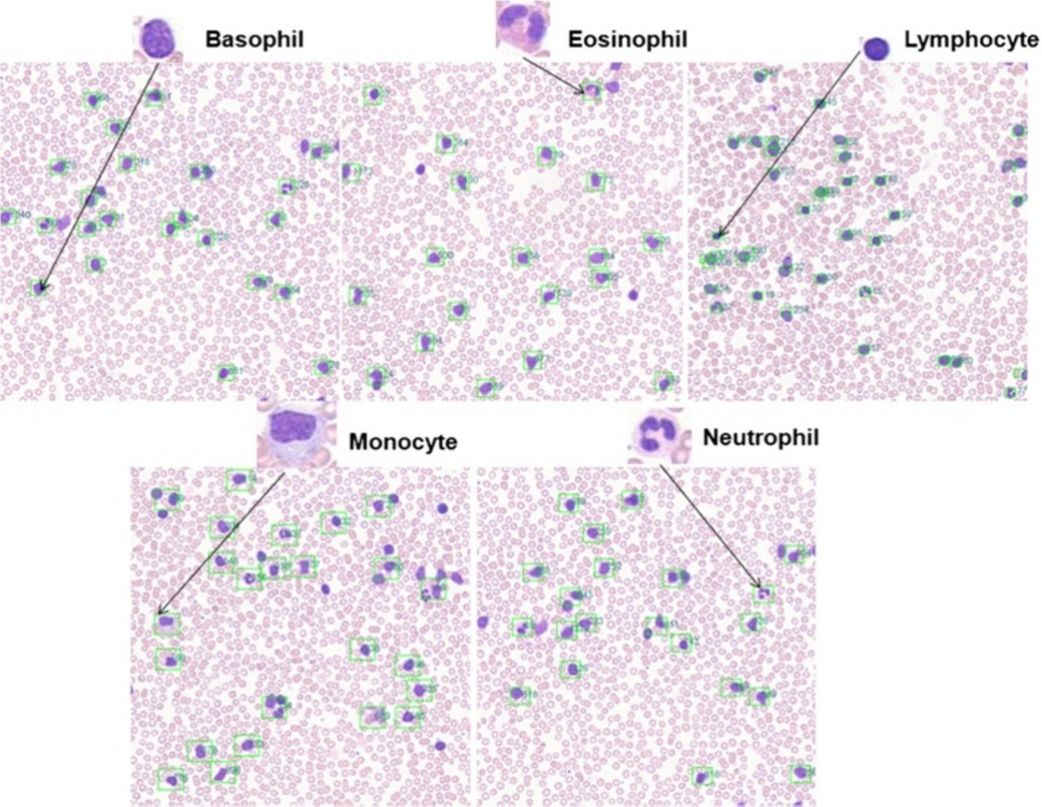
**CBIR results using different classes of leukocytes as query images, including basophil, eosinophil, lymphocyte, monocyte, and neutrophil, respectively.** Here green box labeled regions represent the candidate patches that are similar to the query image patch. Each box has a number to indicate the ranking order of every candidate patch in the dataset. The original sizes of the images were adjusted to fit in the figure.

**Figure 6:**
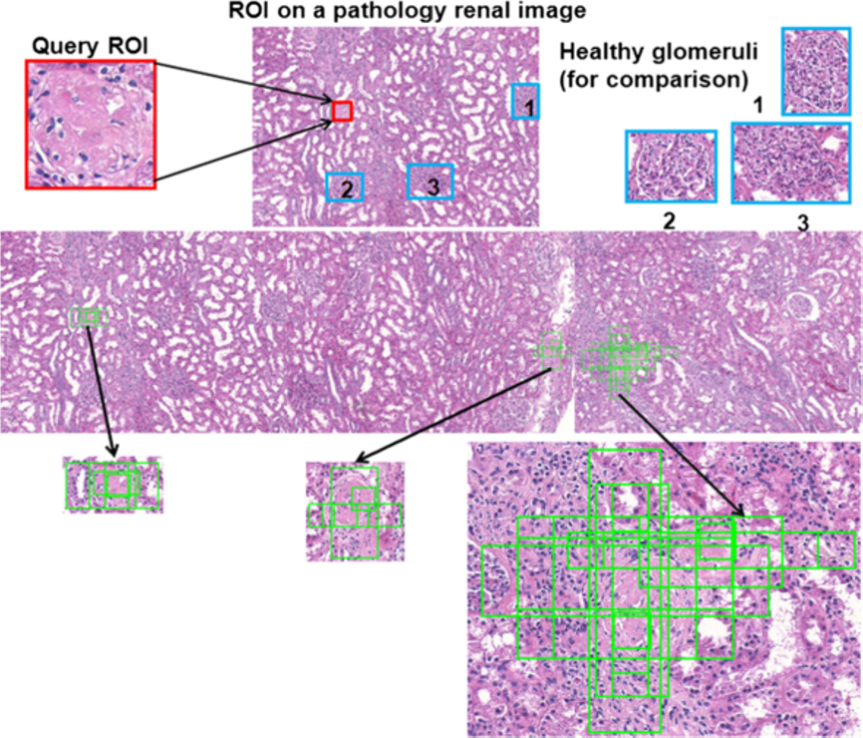
**An example of top 10% CBIR results for a necrotic glomerulus query image.** Red box labeled regions indicate the query image. Blue box labeled regions represent the healthy glomeruli for comparison. Green box labeled regions denote the top 10% ranked retrieved patches, which include multiple scaled regions at 1/2, 1, 2, 3, and 4 times of the original size of the query image. The original sizes of the images were adjusted to fit in the figure.

A dual-processor system based on Intel Xeon E5530@2.4 GHz with 24 GB RAM and 64-bit operating system was used for the CBIR study. Initial CBIR results using two pathology image datasets and different feature comparison are presented below. Figure 4 shows an example of the CBIR three-stage hierarchical searching results using one neutrophil as a query image in a peripheral blood smear dataset acquired using 20× magnification objective. Green box labeled regions represent the candidate patches that are similar to the query image patch. Figure 5 shows CBIR results using different classes of leukocytes as query images, including basophil, eosinophil, lymphocyte, monocyte, and neutrophil, respectively. Green box labeled regions represent the candidate patches that are similar to the query image patch. Each box has a number to indicate the ranking order of every candidate patch in the dataset. Figure 6 shows an example of CBIR results for a necrotic glomeruli query image using a testing dataset containing multi-scale regions at 1/2, 1, 2, 3, and 4 times of the original size of the query image. Red box labeled regions indicate the query image. Blue box labeled regions represent the healthy glomeruli for comparison. Green box labeled regions represent the top-ranked 10*%* of retrieval patches of the 32 randomly selected regions (5024×3504 pixels) cropped from whole-slide scanned images.

By varying the number of rings ∈ [ 2,3,5,10,15] in the hierarchical searching, the performance of CBIR is summarized as follows. For imaged peripheral blood smears, all five classes of leukocytes were correctly retrieved using three inner rings of the HAH. For imaged renal glomeruli, as the number of rings increased to 10, all necrotic glomeruli were correctly retrieved. With an increase of the number of the rings, the computational time also increased. The number of rings was shown to be dependent upon the complexity of the dataset.

**Figure 7:**
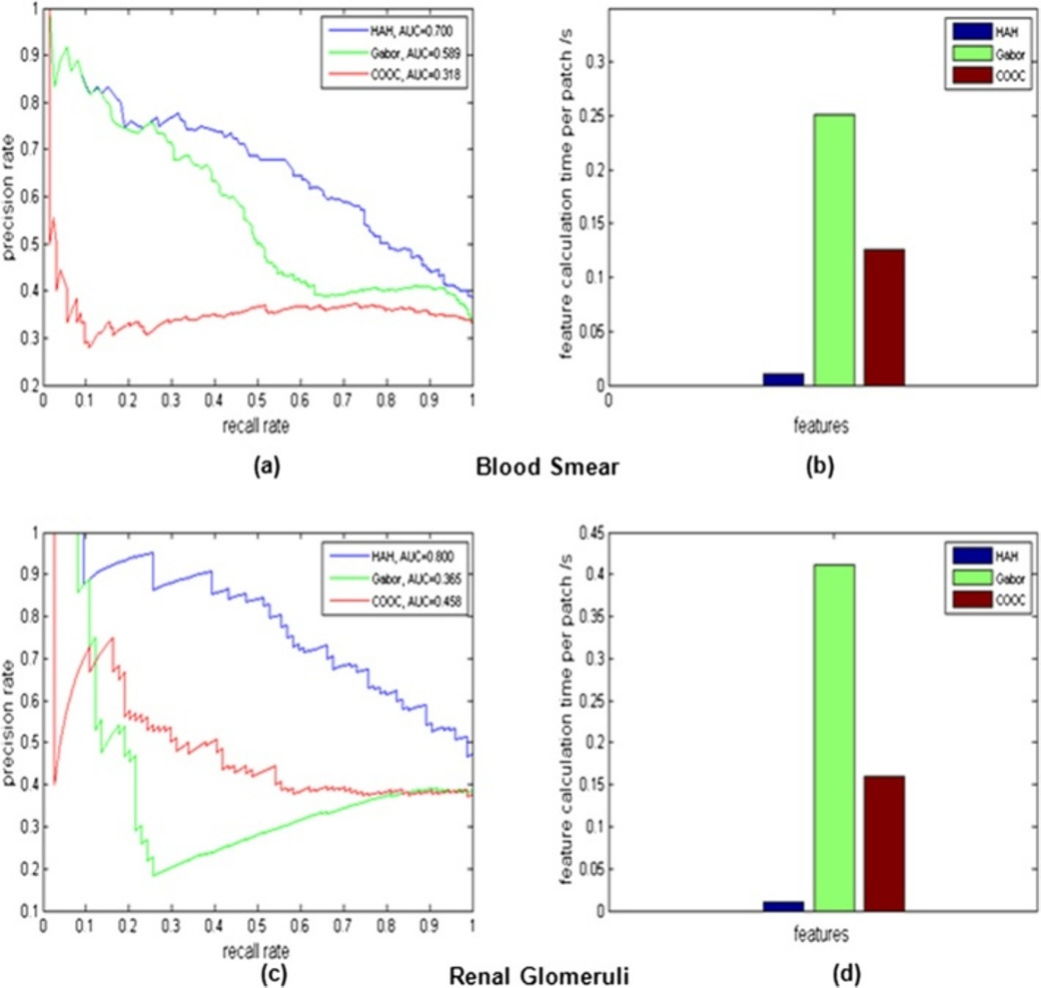
**Local feature comparison using HAH, Gabor wavelet and COOC texture features. (a)** Precision-recall curves of CBIR results using HAH, Gabor wavelet, and COOC texture features on peripheral blood smears. **(b)** Average of feature calculation times per image patch using HAH, Gabor wavelet, and COOC texture features on peripheral blood smears. **(c)** Precision-recall curves of CBIR results using HAH, Gabor wavelet, and COOC texture features on renal glomeruli images. **(d)** Average of feature calculation times per image patch using HAH, Gabor wavelet, and COOC texture features on renal glomeruli images.

For local feature comparison, image retrieval was performed on the same datasets with the same query images using HAH, Gabor wavelet, and COOC texture features. Figure 7(a) and (b) show precision-recall curves and average of feature calculation times using peripheral blood smear images, respectively. Figure 7(c) and (d) show precision-recall curves and average of feature calculation times using renal glomeruli images, respectively. The area under a curve (AUC) value of each feature for peripheral blood smear images and renal glomeruli images are shown in Figure 7(a) and (c), respectively. The average of feature computation times are shown in Figure 7(b) and (d). Based on these experiments, it is clear that HAH feature outperforms Gabor wavelet and COOC texture features with respect to both speed and accuracy.

### Validation of relevance feedback

To evaluate the performance of the dual-similarity relevance feedback algorithm, both peripheral blood smear and multi-scale renal datasets were used. Table 1 summarizes the numbers of relevant/non-relevant images within initial top retrieved 100 images for peripheral blood smear and renal glomeruli datasets, which were labeled by two pathologists with consensus. In general, the percentages of basophils and eosinophils in a given specimen are quite small (e. g., less than 1*%* and 4*%* in our dataset as shown in Table 2). In addition, they can be accurately retrieved as we show in Table 1. Due to this reason, only neutrophils, monocytes, and lymphocytes were utilized for relevance feedback analysis. In those experiments, we applied relevance feedback on the first 100 initial retrieved image patches because this number was sufficient to retrieve all similar cases in the datasets.

**Figure 8:**
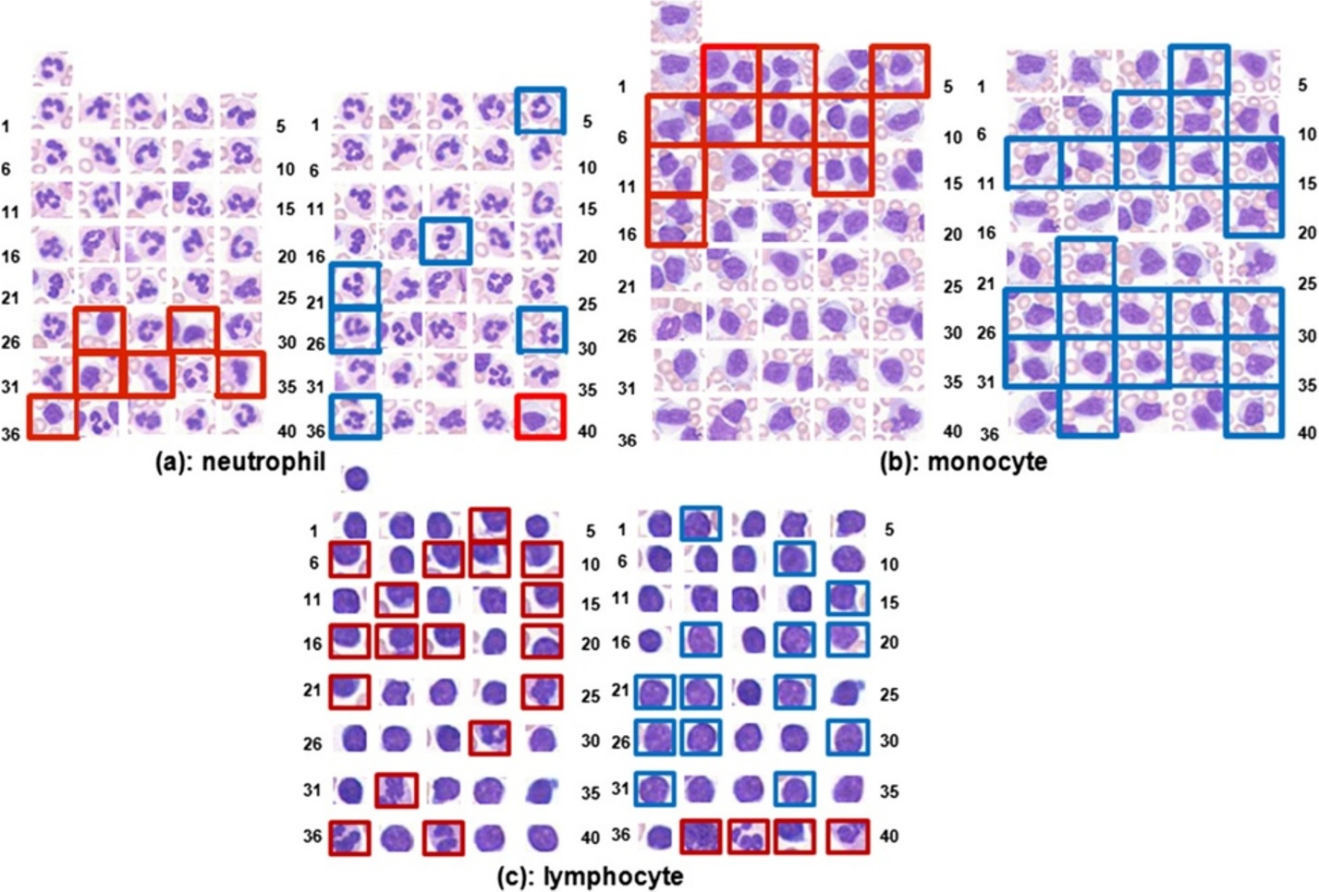
**Top ranked patches before and after relevance feedback of three classes of leukocytes ((a) neutrophil, (b) monocyte, and (c) lymphocyte).** Patches with red rectangles represent the incorrect results (negative examples), and blue rectangles denote the correct results (positive examples), which were re-assigned to higher rankings through the relevance feedback process. The original sizes of the images were adjusted to fit in the figure.

**Figure 9:**
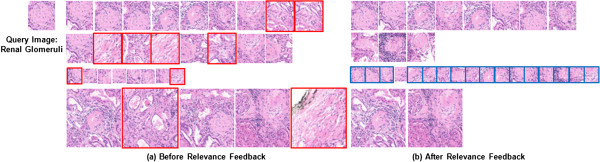
**Top ranked patches before and after relevance feedback of the renal glomeruli dataset.** Patches with red rectangles represent the incorrect results (negative examples), and blue rectangles represent the correct results (positive examples), which were re-assigned to higher rankings through the relevance feedback process.

The original query images, initial top retrieval results, and re-ranked results after relevance feedback are showed in Figures 8 and 9 for blood smear and renal datasets. In both figures, image patches with red rectangles represent the incorrect results (negative examples), and the blue ones represent the correct results (positive examples), which were re-assigned to higher ranking after relevance feedback. For the retrieval results of leukocyte image datasets, the ranking of many correct patches were increased from their initial ranking after relevance feedback. Relevance feedback corrected for 5/6 of the incorrect retrieval patches and increased the ranking for 7 patches from the lower ranking (with initial ranking between 41 and 100) in the neutrophil dataset. This procedure also amended all 10 incorrect patches, and increased ranking for 23 patches in the monocyte dataset. This procedure eliminated all 4 incorrect patches, and increased ranking for 35 patches in the lymphocyte dataset. For the renal dataset, the relevance feedback procedure successfully increased the ranking for all of the 9 correct patches of multi-scale renal dataset shown in Figure 9.

**Figure 10:**
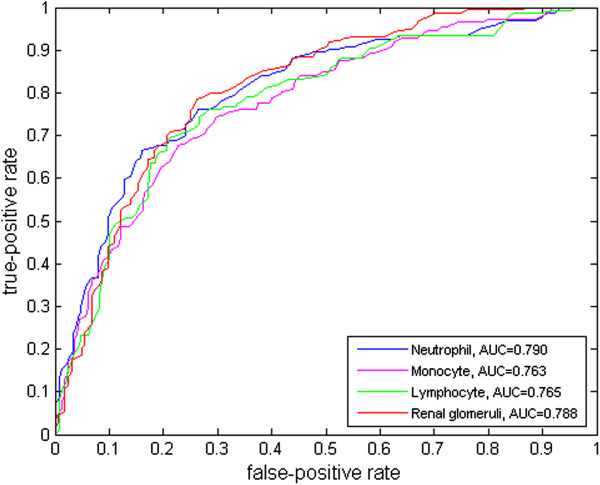
**The ROC curves of the dual-similarity relevance feedback using the peripheral blood smear image dataset (neutrophil, monocyte, and lymphocyte), and the renal glomeruli dataset.**

Ten-fold cross-validation was applied to evaluate the performance of the proposed dual-similarity relevance feedback with receiver operating characteristic (ROC) curves for both peripheral blood smear and renal datasets. The ROC curves after applying relevance feedback on the peripheral blood smear and multi-scale renal datasets are shown in Figure 10.

**Figure 11:**
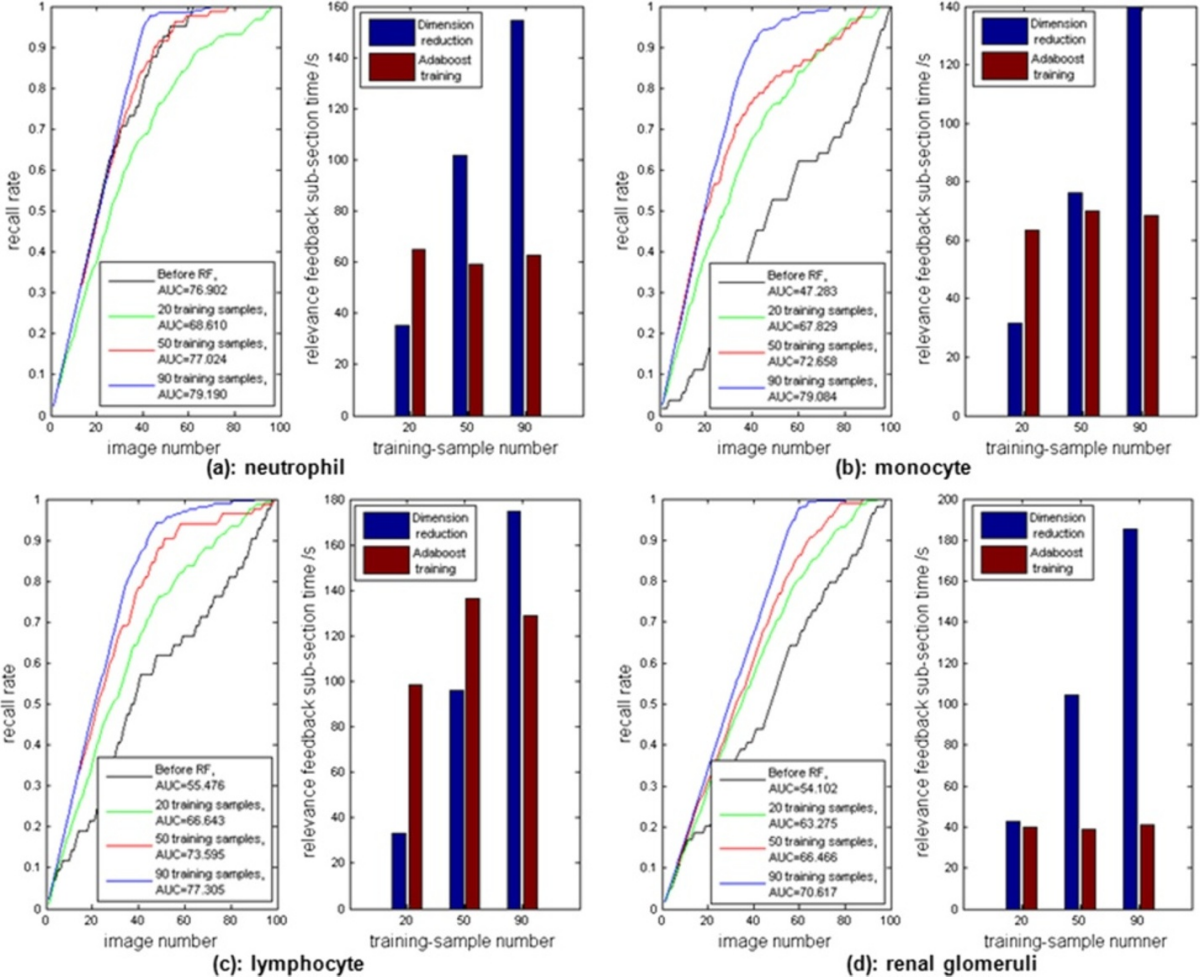
**The recall curves after relevance feedback (RF) and their calculation times using the peripheral blood smear image dataset ((a) neutrophil, (b) monocyte, and (c) lymphocyte)), and (d) the renal glomeruli dataset.** The numbers of training samples were 20, 50 and 90.

Another measures of performance for the proposed relevance feedback are the recall rate and processing speed. The relevance feedback (RF) calculation time includes feature vector dimension reduction and Adaboost classifier training. The numbers of training samples were 20, 50, and 90, and the training samples were randomly selected from the datasets. Based on Figure 11, the values of area under recall curves increased as the number of training samples increased for three leukocytes ((a) neutrophil, (b) monocyte, and (c) lymphocyte), and (d) renal glomeruli. The recall rate after RF for neutrophils (a) using 20 training samples was no better than the result before RF. This was because the original retrieval process already provided a good performance. As the value of area under recall curve before RF was already 76.902, which was much higher than the rest of cases ((b) monocyte, (c) lymphocyte, and (d) renal glomeruli). In this specific case, there was no significant improvement using RF in a small training set (e.g., 20 training samples). However, RF significantly improved the recall rate in larger training sets (e. g., 50 and 90 training samples). In general, the values of area under recall curves were significantly increased after RF with the number of training samples increased.

**Table 1:** **Numbers of relevant/non-relevant images within top 100 initially retrieved images for peripheral blood smear and renal glomeruli datasets, which were labeled by two pathologists with an agreement**

Dataset	# of relevant images	# of non-relevant images
Neutrophil	41	59
Monocyte	53	47
Lymphocyte	42	58
Eosinophil	9	91
Basophil	1	99
Renal tissue	59	41

**Table 2:** **Percentage of various leukocytes in adults approximately**

Various leukocytes	From %	To %
Neutrophil	60	70
Monocyte	3	8
Lymphocyte	20	25
Eosinophil	2	4
Basophil	0.5	1

### Acceleration of CBIR using CometCloud

We conducted experiments to test the performance of CBIR using CometCloud. For HAH, we evaluated two leukocytes query images against a dataset of 925 peripheral blood smear images. In the case of CBIR using multi-scale image candidate patches, we evaluated two different renal glomeruli query images against a dataset of 32 renal images. All the experiments were repeated three times to obtain average results.

During the experiments, the input data were initially located on a single site, the required files were transferred as needed. However, once a file was transferred to a remote site, it was locally staged to minimize the amount of data transferred across sites, especially when multiple tasks require the same input data. To address this issue, a pull model was used where workers request tasks when they become idle. In this way, the workload was uniformly distributed across all workers to address the load imbalance.

To accommodate the CBIR algorithms, we federated various resources including HPC clusters and clouds. In particular, we federated a HPC cluster at Rutgers (a Dell Power Edge system with 256 cores in 8-core nodes - “Dell” hereafter), a SMP machine at Rutgers (64 cores - “Snake” hereafter), and 40 large instances from OpenStack [62] (“FutureGrid”, hereafter), which is a cloud similar to Amazon EC2. Currently we are exploiting the inherent task parallelism of the problem, which means that we can divide the algorithm into smaller sub-modules and execute each module independently. This provides a linear scalability as long as we have more tasks than computational cores.

**Figure 12:**
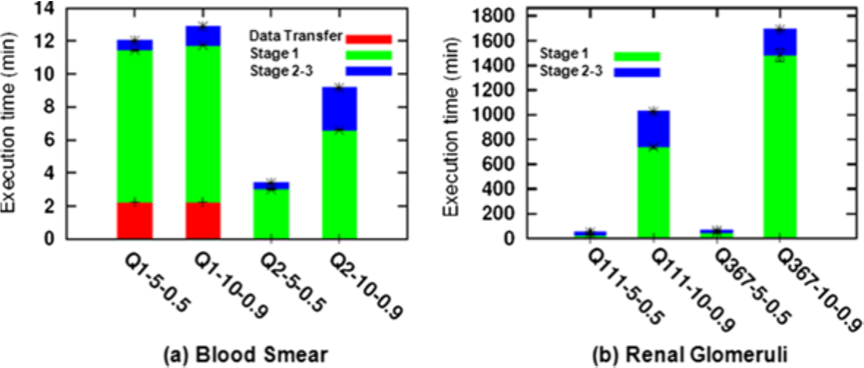
**The execution time of hierarchical searching process using (a) peripheral blood smear dataset, and (b) renal glomeruli dataset, with different combinations of number of the HAH rings and the percentage of overlapping.**

Figure 12 presents a summary of the execution time of the proposed hierarchical searching algorithm using two representative peripheral blood smears and a multi-scale renal glomeruli dataset while varying the parameters, respectively. The results illustrate average values, including error bars showing their associated variabilities. Please note that the *Y*-axes in the sub-figures represent different scales. The figure also demonstrates the execution time of each stage and the time required to transfer the images for processing. Since the image transfer time represents a small fraction of the total execution time (i.e., from a few seconds to a 2–3 minutes depending on the configuration), in our current implementation we copy the images sequentially from a central repository. The execution time varies depending on the algorithm we used, the query and dataset images, and the configuration (e.g., 90*%* overlapping takes longer than 50*%* overlapping). The fraction of time spent on each stage of the hierarchical searching is shown in Figure 12.

**Figure 13:**
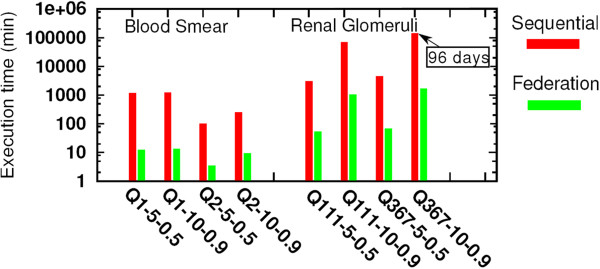
**The execution time of sequential and federated infrastructure using peripheral blood smear dataset and renal glomeruli dataset with different combinations of number of the rings and the percentage of overlapping.** Here the Y-axis is in a logarithmic scale.

**Figure 14:**
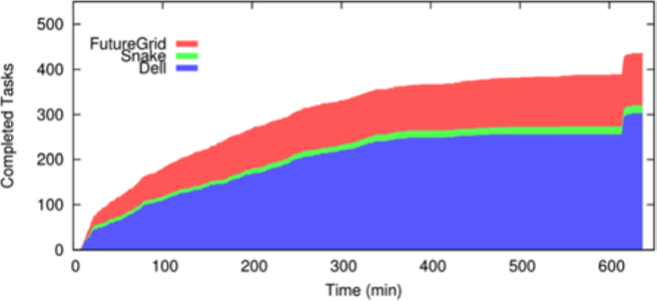
**The number of completed tasks over time when testing the CBIR algorithm using the renal glomeruli dataset.** The area under “FutureGrid” represents the contribution from the cloud resources.

Figure 13 compares the execution time of different configurations using a single system and federated cyber-infrastructure. We observe an average acceleration of 70-fold with a maximum of 96-fold. This is achieved by elastically using multiple resources as discussed below. Figure 14 shows the contribution of the FutureGrid cloud to the execution of the multi-scale algorithm. Cloud resources significantly accelerate the execution of the algorithm. During stages with lower parallelism (e.g., last minutes of the execution), computation can be performed using local HPC resources and cloud resources can be released to reduce operational costs.

**Figure 15:**
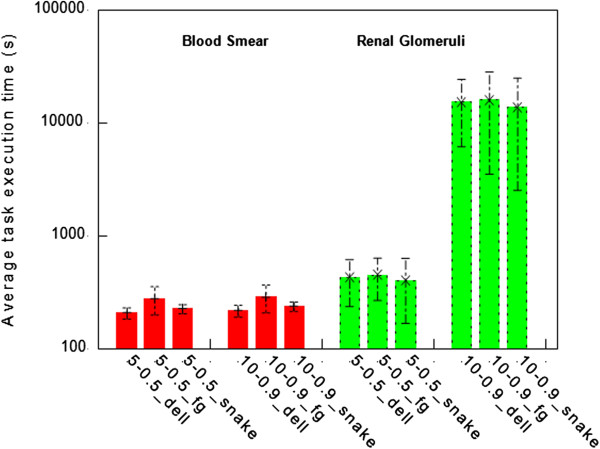
**The average task execution time per platform using the peripheral blood smear and renal glomeruli datasets with different combinations of number of the rings and the percentage of overlapping.** Here “FutureGrid” is abbreviated as “fg”.

**Figure 16:**
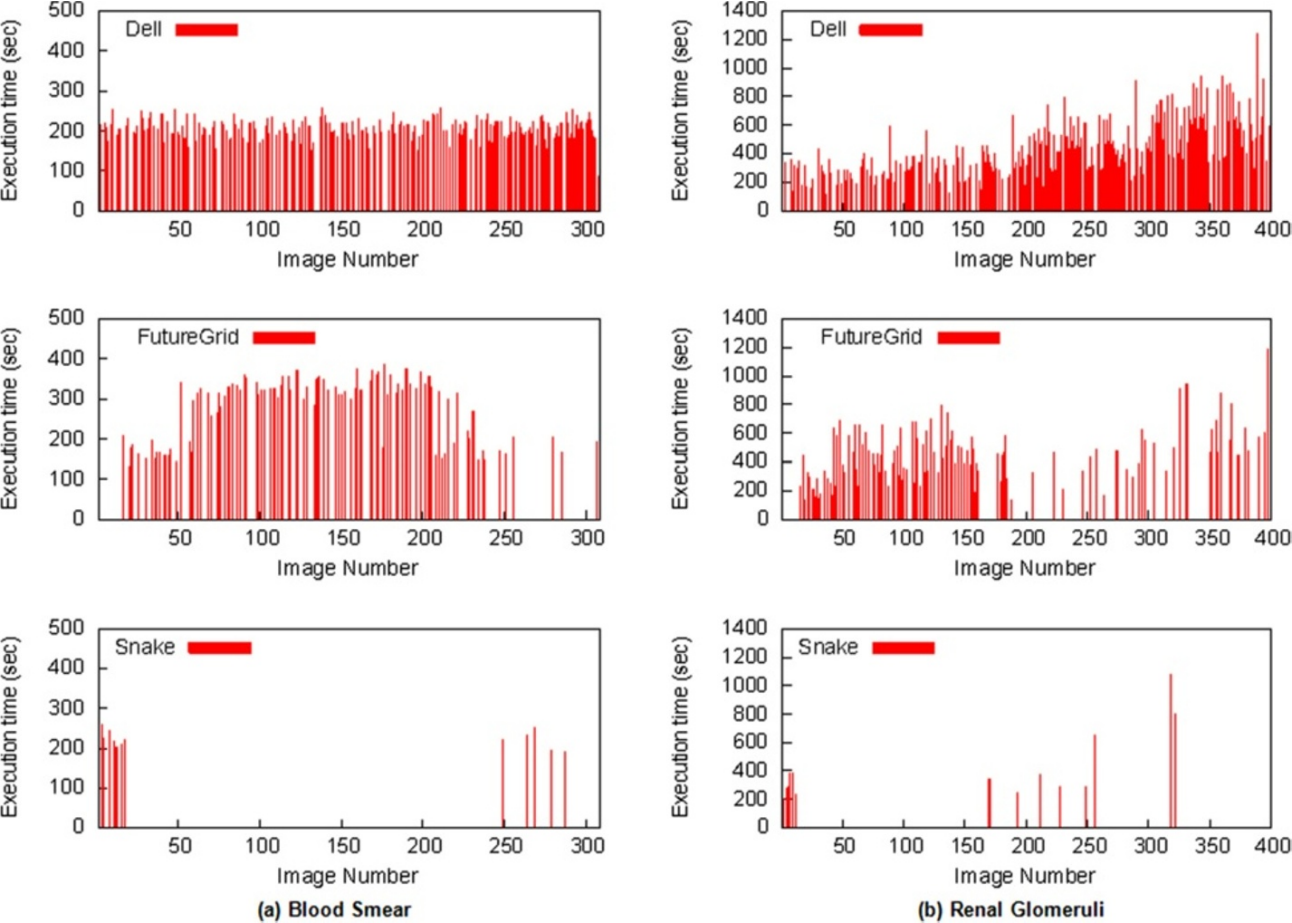
**The execution time per task using (a) the peripheral blood smear dataset and (b) the renal glomeruli dataset.**

The variability of the execution time of different tasks is shown in Figures 15 and 16. Figure 15 shows the average task execution time and variability using different configurations. The variability of task execution time is heterogeneous and depends on the configurations and the machine. In general, the longer the execution takes, the larger the variability. Figure 16 shows that the execution time of individual task is relatively heterogeneous. It also demonstrates that the distribution of tasks among different federated resources depends on the number of cores available in each platform (e.g., one of the cores, snake, runs only a few tasks). The results show that the parallelization of CBIR at the image level can dramatically reduce the overall computational time.

## Conclusion

In this paper, we present a set of newly developed CBIR algorithms and demonstrate its application on two different pathology applications, which are regularly evaluated in the practice of pathology. The experimental results suggest that the proposed CBIR algorithm using sequential HAH searching follows a progression which parallels to the same logical steps as ever invoked when physicians review digital pathology images. During the review process, the pathologist typically begins by first identifying gross locations of potential regions of interest (coarse searching in the proposed algorithm) before executing the more refined stages (fine searching in the proposed algorithm) to examine the detailed morphometric characteristics.

For the peripheral blood smear study, we tested performance using a range of different leukocytes and experimentally showed the reliable performance of the CBIR algorithm. The success of the proposed CBIR algorithm in identifying neutrophils suggests further exploration of the HAH feature in detecting abnormal or hypersegmented neutrophils, which are indicators of megaloblastic anemia and potential risk of gastric cancer. Similarly, a pathologist’s assessment of normal vs. diseased glomeruli in renal biopsies is often used as an indicator of overall kidney health, such as, the determination of graft function from pre-transplantation biopsies [55]. Assisted by the proposed CBIR algorithm, physicians and researchers can quickly review a digital biopsy to evaluate the proportion of ischemic or necrotic glomeruli within a given field to quickly assess whether an incoming specimen is suitable for transplantation or not. This type of review can have multiple applications, such as, determining whether a rejection of the organ might occur by identifying areas of focal and segmental glomerulosclerosis [63]. Currently, our algorithm requires some external feedback to optimize the search. We are exploring different ways of automatizing this process by applying machine learning techniques. On the other hand, although the proposed hierarchical searching has significantly improved the retrieval speed, it is still a computational demanding procedure. Therefore, we are exploring new ways of exploiting parallelism to speed-up this process.

We present a generalizable cloud-enabled CBIR algorithm that can be extended to a wide variety of applications. Because of the computational requirements needed for retrieving whole-slide scanned images, we explore the use of federated high performance computing (HPC) cyber-infrastructures and clouds using CometCloud. Comparative results of HPC versus standard computation time demonstrate that the CBIR process can be dramatically accelerated, from weeks to minutes, making real-time clinical practice feasible. Moreover, the proposed framework hides infrastructure and deployment details and offers end-users the CBIR functionality in a readily accessible manner. We are currently working on improving the utilization of resources by exploit the particular capabilities and capacities of each heterogeneous resource, e.g., switching between the usage of the original CBIR implementation in MATLAB (The MathWorks, Natick, MA) when licenses are available or a parallel implementation using graphic processing unit (GPU) and many-core architectures in cases where resources with accelerators are available.
